# Microstructural Dependence of the Impact Toughness of TP316H Stainless Steel Exposed to Thermal Aging and Room-Temperature Electrolytic Hydrogenation

**DOI:** 10.3390/ma17174303

**Published:** 2024-08-30

**Authors:** Ladislav Falat, Lucia Čiripová, Viera Homolová, Miroslava Ďurčová, Ondrej Milkovič, Ivan Petryshynets, Róbert Džunda

**Affiliations:** 1 Institute of Materials Research, Slovak Academy of Sciences, Watsonova 47, 04001 Košice, Slovakia; lfalat@saske.sk (L.F.); vhomolova@saske.sk (V.H.); miroslava.durcova@tuke.sk (M.Ď.); omilkovic@saske.sk (O.M.); ipetryshynets@saske.sk (I.P.); rdzunda@saske.sk (R.D.); 2 Faculty of Materials, Metallurgy and Recycling, Technical University of Košice, 04200 Košice, Slovakia

**Keywords:** TP316H stainless steel, isothermal aging, electrolytic hydrogenation, impact toughness, fracture micro-mechanism

## Abstract

This work deals with the effects of two individual isothermal aging experiments (450 °C/5000 h and 700 °C/2500 h) and the subsequent room-temperature electrolytic hydrogen charging of TP316H stainless steel on its Charpy V-notch (CVN) impact toughness and fracture behavior at room temperature. Microstructural analyses revealed that aging at 700 °C resulted in the abundant precipitation of intermediary phases, namely, the Cr_23_C_6-_based carbide phase and Fe_2_Mo-based Laves phase, whereas aging at 450 °C resulted in much less pronounced precipitation of mostly intergranular Cr_23_C_6_-based carbides. The matrix phase of 700 °C-aged material was completely formed of austenitic solid solution with a face-centered cubic (FCC) crystal structure, whereas an additional formation of ferritic phase with a base-centered cubic (BCC) structure was detected in 450 °C-aged material. The performed microstructure observations correlated well with the obtained values of CVN impact toughness, i.e., a sharp drop in the impact toughness was observed in the material aged at 700 °C, whereas negligible property changes were observed in the material aged at 450 °C. The initial, solution-annealed (precipitation-free) TP316H material exhibited a notable hydrogen toughening effect after hydrogen charging, which has been attributed to the hydrogen-enhanced twinning-induced plasticity (TWIP) deformation mechanism of the austenitic solid solution. In contrast, both aging expositions resulted in significantly lowered hydrogen embrittlement resistance, which was likely caused by hydrogen trapping effects at the precipitate/matrix interfaces in thermally aged materials, leading to a reduced TWIP effect in the austenitic phase.

## 1. Introduction

AISI 316H-grade heat-resistant steel represents a high-carbon, nitrogen-free derivative of traditional AISI 316 stainless steel; commercial applications include the fabrication of structural parts for boiler equipment in supercritical thermal power plants, as well as in nuclear power generation plants. Specifically, this material is used in high-temperature applications up to about 700 °C for the construction of superheaters and reheaters that are part of the highly efficient boilers found in most modern coal-fired thermal power plants [1,2,3,4,5]. It is also used or potentially considered for constructing parts found in various nuclear power generation technologies (e.g., in the Generation-IV sodium-cooled fast reactors, advanced gas-cooled fast reactors, lead-cooled fast reactors, molten salt reactors, etc.) in the temperature range of 350 °C–600 °C [6,7,8,9,10,11,12]. Apart from the anticipated thermally induced material degradation, i.e., so-called thermal embrittlement, which gradually affects creep-resistant steels and other heat-resistant alloys during their long-term high-temperature service, material degradation due to hydrogen embrittlement may also be an issue in the boiler equipment of supercritical steam power plants during their regular or accidental shut-downs and when cooled below 150 °C in the presence of residual tensile stresses [13]. Moreover, hydrogen embrittlement tests using hydrogen/deuterium charging under specific experimental conditions are also used in studies simulating the irradiation effects found in various engineering materials [14,15,16,17,18].

The highly alloyed chemical conception of TP316H steel on a Fe-Cr-Ni-Mo-C base means that this material can be considered a non-equiatomic, medium-entropy alloy [19]. Thanks to the high content of austenite-stabilizing nickel in the TP316H material, its matrix phase is primarily formed of austenitic (γ-phase) solid solution with a face-centered cubic (FCC) crystal structure in as-produced material condition. The high Ni content in austenitic stainless steels is necessary to compensate for the influence of ferrite stabilizing elements (e.g., Cr, Mo, and Si) and to suppress the formation of undesirable topologically close-packed (TCP) intermetallic phases like the FeCr-based σ-phase and Fe_2_Mo-based η-phase, i.e., the Laves phase [20]. The high chromium content gives the alloy good oxidation and corrosion resistance in various environments, even at high temperatures [21]. Chromium forms Cr_23_C_6_-based carbides (also denoted as M_23_C_6_, where M = Cr, Fe, and Mo), which are the most important strengthening precipitates for the creep resistance of non-stabilized austenitic steels [22,23,24]. Molybdenum increases the creep resistance via intensive solid-solution hardening; however, it also promotes the formation of unfavorable embrittling phases like σ-phase, η-phase, and χ-phase (Fe,Ni)_36_Cr_12_Mo_10_ [20]. The increased carbon content not only contributes to the increased creep strength via the formation of the above-mentioned Cr_23_C_6_-based carbides but it also suppresses the precipitation of undesirable phases like δ-ferrite, σ-phase, η-phase, and χ-phase [20]. The results of numerous research studies focused on 316-type stainless steels and especially on 316H steel are widely available in the literature, e.g., [21,22,23,24,25,26]. However, due to small compositional variations, e.g., heat-to-heat variations, impurity of content, or other various metallurgical/processing effects, the obtained results regarding microstructure, phase composition, and the properties of 316-type steels may be more or less different. Therefore, it is necessary to study each specific 316-type material individually. Recently published studies [21,27,28,29] about 316H-grade stainless steel have resulted in the following newly obtained findings. In the work of Li et al. [21], the effect of thermal aging on the corrosion behavior of 316H-type stainless steel in molten chloride salt was investigated and it was revealed that thermally aged 316H steel was more resistant to molten chloride salt corrosion than the as-received annealed material. It was found that Cr_23_C_6_-based carbide and η-phase precipitated in 316H steel during 3000 h of aging at 700 °C; the higher corrosion resistance in the molten salt was ascribed to the more thermally stable microstructure of thermally aged material, in which chromium diffusion proceeded more slowly compared to the as-received material, where the intensive nucleation/precipitation of Cr_23_C_6_-based carbide and corrosion took place simultaneously [21]. Pan et al. [27] investigated the effects of strain rate on the tensile and creep-fatigue properties of 316H stainless steel. They revealed that 316H stainless steel exhibited a dynamic strain aging (DSA) effect within a certain range of testing temperatures and strain rates. They reported that the DSA effect increased with the decrease in the strain rate. The observed DSA effect enhanced the initiation of cracks in the grain boundary, resulting in multiple cracks in the sample, while intergranular fracture occurred when the fatigue lines extended to the grain boundaries [27]. He et al. [28] studied the role of grain-boundary ferrite evolution and thermal aging on creep cavitation of 316H-type austenitic stainless steel. They suggested that in ex-service 316H steel that had been thermally aged in the temperature range of 490 °C–530 °C for 65,000 h, the formation and growth of additional Cr_23_C_6_ carbides at the austenite grain boundaries promoted the nucleation of ferrite from the localized chromium-depleted regions of austenite [28]. Most research studies about the impact toughness of 316-type stainless steels are mainly focused on the 316L steel type, i.e., the low-carbon 316 steel variant [29,30,31].

In the present work, heat-resistant TP316H steel was investigated with respect to the superposition effect of prior thermal aging and subsequent electrochemical hydrogen charging on the resulting impact toughness and fracture behavior at room temperature. The obtained results regarding the brittle fracture resistance of the studied material are discussed in correlation with its microstructure characteristics and fracture micro-mechanisms in dynamic loading conditions.

## 2. Experimental Material and Procedures

When received, the TP316H material was in the form of a seamless tube (38 mm in outer diameter, 5.6 mm in wall thickness). Its chemical composition is given in Table 1.

**Figure 1:**
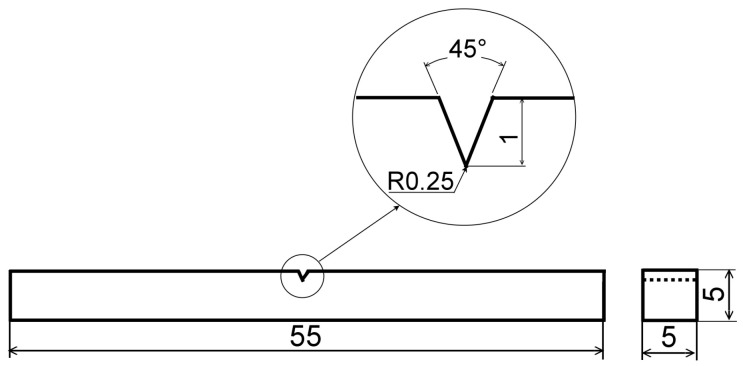
Sub-sized Charpy V-notch (CVN) specimen for the impact bending test. All the dimensions are given in mm.

The individual heat-treated material states, namely, a solution-annealed state (1060 °C/0.5 h/water quench) and two isothermally aged material states (450 °C/5000 h/water quench and 700 °C/2500 h/water quench) were prepared for 55-millimeter-long TP316H tube segments by performing individual heat-treatment procedures using an electric resistance furnace LAC PKE 18/12R (LAC, s.r.o., Rajhrad, Czech Republic). Charpy V-notch (CVN) impact toughness, characterizing the brittle fracture resistance of the studied material under impact loading conditions, was obtained by means of a conventional Charpy pendulum impact test at room temperature [32]. The tests were performed by employing a conventional Charpy pendulum impact tester PSW 30 (VEB Werkstoffprüfmaschinen Leipzig, Leipzig, Germany) and sub-sized CVN test specimens (see Figure 1). 

The sub-sized specimens were prepared by the combination of an electrical discharge machining technique using a spark erosion machine EIR-EMO 2N (Emotek s.r.o., Nove Mesto nad Vahom, Slovakia), and conventional machining (grinding). The CVN impact toughness tests were carried out for all prepared heat-treated material states in conditions without and with subsequent electrochemical hydrogenation. The electrochemical hydrogenation of prepared sub-sized CVN test specimens was carried out at room temperature in a solution of 1 M HCl with 0.05M N_2_H_6_SO_4_ at a current density of 200 A/m^2^ for 24 h. For each material condition, three CVN test specimens were examined and the average CVN impact toughness values were determined. The electrolytic hydrogenation of the test specimens was performed at ambient temperatures using a potentiostat/galvanostat model 173 (Princeton Applied Research, Oak Ridge, TN, USA). The hydrogen embrittlement resistance of individual heat-treated material states was estimated from the corresponding average CVN impact toughness values for non-hydrogenated and hydrogen-charged material conditions. 

The microstructure analyses of the investigated material were performed using a light-optical microscope (LOM) OLYMPUS GX71 (Olympus Corporation, Tokyo, Japan) and scanning electron microscope (SEM) JEOL JSM-7000F (Jeol Ltd., Tokyo, Japan) linked with an energy-dispersive X-ray (EDX) analyzer INCA X-sight model 7557 (Oxford Instruments, Abingdon, Oxfordshire, UK). Standard metallographic procedures were used for the preparation of metallographic cross-sections for microstructural observations. Conventionally prepared metallographic specimens were etched in a solution of “Aqua Regia” (i.e., an acidic solution of concentrated HCl and HNO_3_ acids in a molar ratio of 3:1). Fractographic analyses of the fracture surfaces of broken CVN specimens after impact bending tests were carried out using a scanning electron microscope Tescan Vega-3 LMU (TESCAN Brno, s.r.o., Brno, Czech Republic), enabling the insertion of whole fractured specimens into the SEM working chamber.

All phase equilibria and phase diagram sections of the Fe-Cr-Mn-Mo-Ni-Si-C system, including the prediction of the phase composition of the long-term-aged TP316H material, were determined using the equilibrium thermodynamic calculations in Thermo-Calc software (version S, Thermo-Calc Software AB, Solna, Sweden), employing the non-commercial thermodynamic database STEEL16 developed by Dr. Aleš Kroupa (IPM, CAS, Brno, Czech Republic). Experimental determinations of the phase compositions were carried out by X-ray diffraction (XRD) and electron back-scattered diffraction (EBSD) with a Philips X’Pert Pro diffractometer (Panalytical B.V., Almelo, The Netherlands) in Bragg–Brentano geometry, with Cu-Kα radiation and the EBSD detector Nordlys-I ( Oxford Instruments plc, Abingdon, UK), respectively. The XRD pattern was used for phase identification and refined by the Rietveld method for the volume ratio calculation of the major phases using the “Materials Analysis Using Diffraction” (MAUD) software (Version: 2.92, Luca Lutterotti, University of Trento, Trento, Italy) [33,34]. The two major expected phases, i.e., austenite and ferrite, were identified from the XRD pattern in the steel sample in such a way that the phase models ICDD-04-002-8935 and ICDD-04-006-6420 were used for refinement, respectively. This refinement was focused on obtaining the volume ratio of phases; therefore, only the lattice parameters, isotropic strain-size values, and exponential harmonic texture were used for the refinement procedure. 

EBSD phase mapping was used for the detection of the major phases of the matrix, whereas minor phases, i.e., the secondary phase precipitates, were identified by means of Kikuchi diffraction patterns obtained from the local point diffraction analyses of the investigated precipitate particles. The EBSD analyses were performed on a drawing direction plane of prepared metallographic specimens of aged TP316H materials and the obtained EBSD data were processed using the CHANNEL-5, HKL software package (service pack 7, HKL technology A/S, Hobro, Denmark). Crystallographic data (unit cell parameters, space group, and the chemical composition of the phase and atom Wyckoff position in a crystal lattice) for individual anticipated phases were taken from the EBSD software database and the database for “The Materials Project” [35].

## 3. Results and Discussion

### 3.1. The Effect of Thermal Aging on Microstructure and Phase Composition

**Figure 2:**
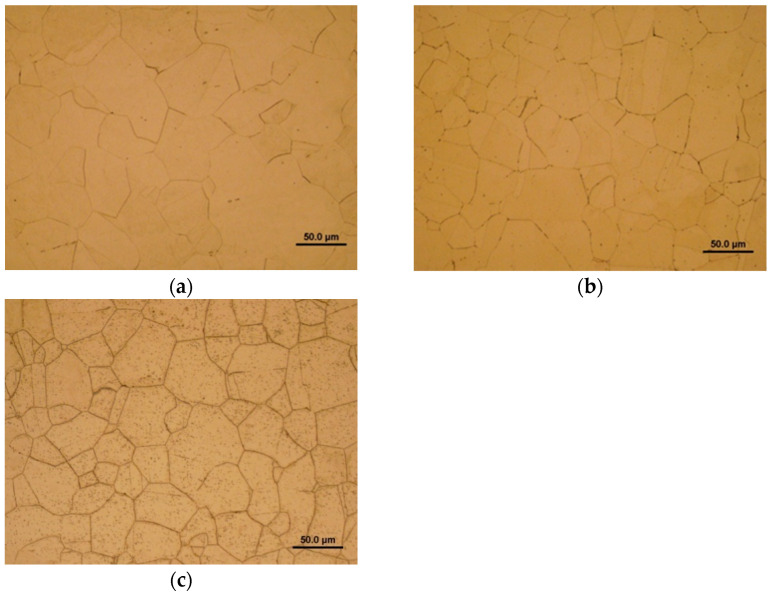
Light-optical microscopic images of TP316H steel under various heat-treated material conditions: (**a**) “1060 °C/0.5 h”, (**b**) “450 °C/5000 h”, (**c**) “700 °C/2500 h”.

First, the effects of high-temperature aging on the microstructure and phase composition of TP316H steel were investigated. Figure 2 shows light-optical microscopic images of the studied material in three heat-treated material states, namely, the initial solution-annealed state “1060 °C/0.5 h” (Figure 2a) and two isothermally aged material states, i.e., “450 °C/5000 h” (Figure 2b) and “700°C/2500 h” (Figure 2c).

The similar solution-annealed material state of the TP316H material was characterized in detail by means of XRD and EBSD techniques in our previous study [36]. Generally, regardless of some impurity features like some rare occurrences of MnS non-metallic inclusions, a single-phase austenitic polygonal grain structure with face-centered cubic (FCC) crystal structure was also revealed for the current initial material conditions (Figure 2a). Figure 2b depicts the 450 °C thermally aged microstructure of TP316H material, showing some slight precipitation on the grain boundaries. The precipitates, presumably those of the Cr_23_C_6_-based carbides, are formed as a consequence of Cr and C intergranular segregation, subsequently resulting in Cr_23_C_6_ carbide precipitation thanks to rapid carbon diffusion and its higher chemical affinity to chromium than to iron [37]. These precipitates are further identified by means of SEM/EDX and EBSD analyses. Figure 2c shows the 700 °C thermally aged microstructure of TP316H material, exhibiting the abundant precipitation of secondary phase precipitates on both the high-angle grain boundaries and annealing twin boundaries, as well as within the grain interiors. These observations were not expected since, with the decreasing temperature, the solid solubility of alloying elements in the alloy also decreases, which gives rise to more pronounced precipitation at lower temperatures than at higher temperatures. However, the obtained findings show quite the opposite result, i.e., a larger amount of precipitation of the secondary phase precipitates was observed at higher temperatures, whereas a much smaller amount of precipitation was revealed at lower temperatures. This finding is caused by the prevailing kinetic factor over the thermodynamic factor, due to a likely insufficient aging time at a lower temperature to reach thermodynamic equilibrium.

**Figure 3:**
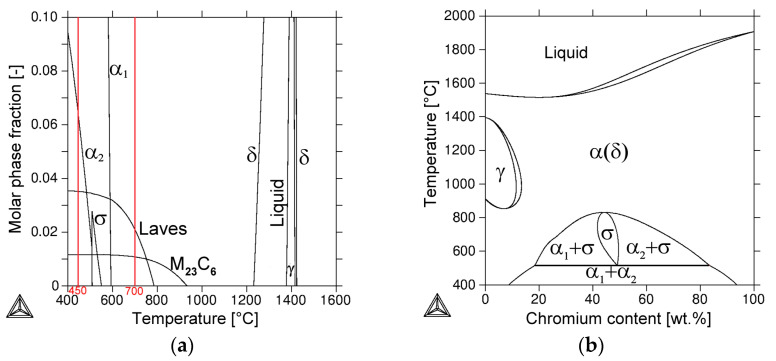
Thermodynamic predictions of stable phases using the software Thermo-Calc: (**a**) Temperature dependence of molar phase fractions of stable phases in TP316H steel; the red vertical lines are isotherms at 450 °C and 700 °C indicating phase compositions of studied material states, (**b**) Binary Fe-Cr phase diagram showing the origin of the occurrence of two immiscible solid solution phases, i.e., α_1_ (Fe-rich) and α_2_ (Cr-rich), at low temperatures.

Prior to detailed experimental studies focused on the phase analyses of individual isothermally aged material states, thermodynamic predictions of the stable phases were carried out by means of Thermo-Calc equilibrium calculations (see Figure 3). Due to the above experimental findings being related to the currently used aging conditions, the results of the performed thermodynamic calculations should be considered only qualitatively.

The temperature dependence of molar phase amounts of thermodynamically stable phases in TP316H steel is shown in Figure 3a. The intersections of black phase lines with red isothermal lines correspond to thermally aged material conditions and determine the calculated phase fractions of individual equilibrium phases in TP316H material at 450 °C and 700 °C. As can be seen in Figure 3a, the predicted phases for material aged at 450 °C include the γ (FCC) austenitic solid solution, α_1_ (BCC), and α_2_ (BCC) solid solutions, Cr_23_C_6_-based carbide, and the Fe_2_Mo-based Laves phase. In contrast, the predicted phases for material aged at 700 °C include the γ (FCC)-based austenitic solid solution, Cr_23_C_6_-based carbide, and Fe_2_Mo-based Laves phase. The origin of the occurrence of two BCC-structured solid-solution phases, i.e., α_1_ (Fe-rich) and α_2_ (Cr-rich), in TP316H material at lower temperatures can be explained by the existence of spinodal decomposition phase transformation in the binary Fe-Cr phase diagram (Figure 3b), which represents a partial thermodynamic sub-system of the multi-component system of TP316H material.

**Figure 4:**
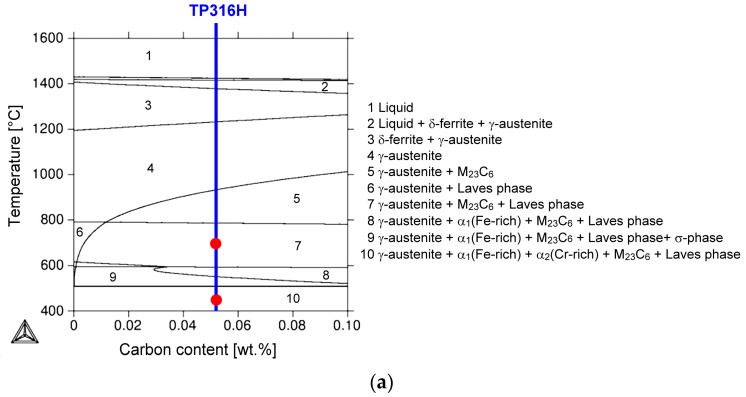

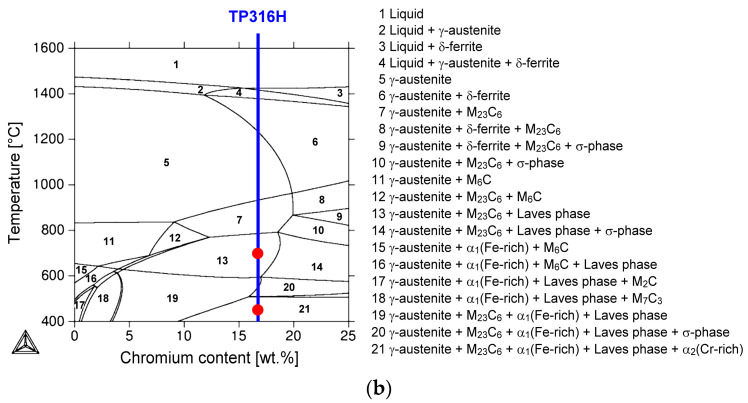
Equilibrium phase diagrams of the Fe-Cr-Mn-Mo-Ni-Si-C system, calculated using the Thermo-Calc software: (**a**) isoplethal section of Fe-16.76Cr-1.77Mn-2.05Mo-11.13Ni-0.51Si-C system with Fe/C content ratio variation and (**b**) isoplethal section of the Fe-Cr-1.77Mn-2.05Mo-11.13Ni-0.51Si-0.052C system with Fe/Cr content ratio variation. The red circles depict the positions of the TP316H material within the corresponding phase fields at the specified temperatures.

The complexity of the thermodynamic phase relations in the multi-component alloy TP316H is also demonstrated on two selected isoplethal sections in Figure 4.

The isoplethal sections of the Fe-Cr-Mn-Mo-Ni-Si-C system were calculated for set variations of carbon (Figure 4a) and chromium (Figure 4b) since these chemical elements are considered crucial for the formation of secondary (intermediary) phases, such as carbides and topologically close-packed (TCP) phases (i.e., intermetallics) in the studied multi-component material. The amount of C (Figure 4a) and Cr (Figure 4b) is changed in the calculations at the expense of the amount of Fe. The other elements are kept constant and correspond to the quantities in the studied steel. The individual phase transformations, depending on temperature, in the TP316H material can be demonstrated by blue-colored linear vertical isopleths at 0.052 wt.% C and 16.76 wt.% Cr, shown in Figure 4a and Figure 4b, respectively. The phase compositions of TP316H material at the studied temperatures (450 °C and 700 °C) are depicted within their corresponding phase fields by the red circles in Figure 4.

**Figure 5:**
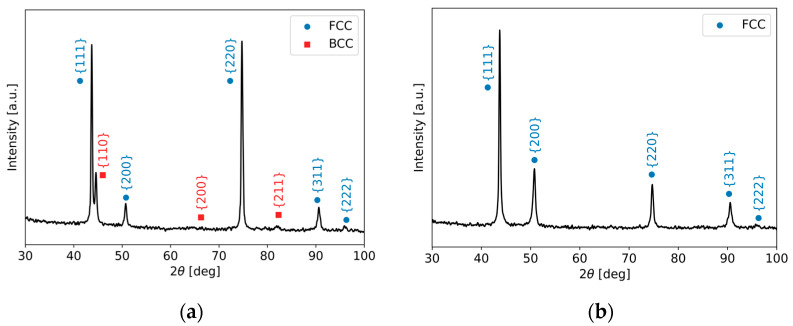
XRD patterns of TP316H material after long-term aging is performed: (**a**) “450°C/5000 h” and (**b**) “700°C/2500 h”.

Next, detailed experimental phase analyses of individual isothermally aged material states were performed by XRD, SEM used with EDX, and EBSD techniques. Figure 5 shows XRD patterns corresponding to both the studied isothermally aged material conditions of TP316H material. In agreement with the performed thermodynamic calculations, the 450 °C thermally aged material state shows both the ferrite and austenite major phases, whereas the 700 °C thermally aged material exhibits only a pure austenitic matrix. The obtained quantitative XRD results were 17% ferrite and 83% austenite for the 450 °C thermally aged specimen. The minor secondary phases, such as the Cr_23_C_6_-based carbide and Fe_2_Mo-based Laves phase, were not detected by the performed XRD measurements, due to the very low phase amounts of these phases (i.e., below the XRD detection limit of about 5% of a phase within the analyzed specimen), as shown previously in Figure 3.

**Figure 6:**
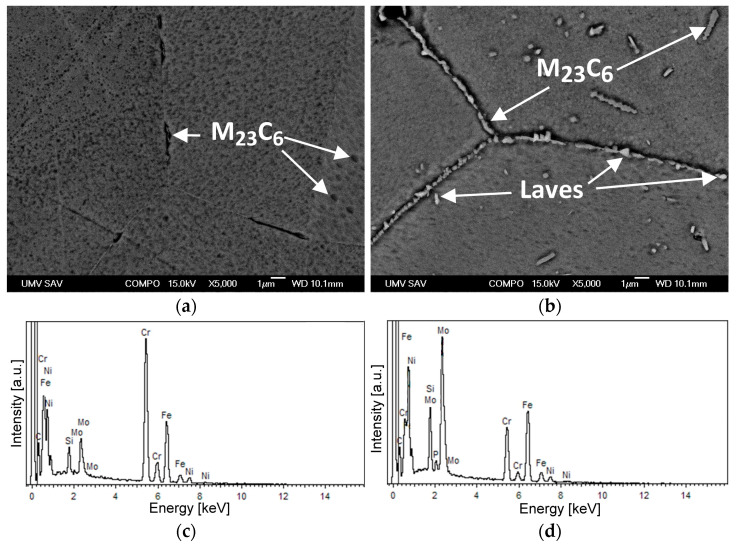
SEM analyses of the studied TP316H material depicting detailed back-scattered electrons (BSE) in a contrast visualization of thermally aged microstructures at “450 °C/5000 h” (**a**) and “700 °C/2500 h” (**b**), with the typical EDX spectra of secondary phase precipitates of Cr_23_C_6_-based carbide (**c**) and Fe_2_Mo-based Laves phase (**d**). The enhanced pitting in the 450 °C thermally aged specimen is probably related to the partial decomposition of an FCC-structured γ matrix into BCC-structured immiscible solid solutions (α_1_, α_2_), with different etching behaviors.

The following investigation of the microstructure and phase composition of the thermally aged material states of the studied TP316H steel was carried out using SEM observations and EDX chemical micro-analyses of the minor secondary phases in prepared metallographic cross-sections (Figure 6).

According to the performed thermodynamic predictions (Figure 3a and Figure 4), both the 450 °C and 700 °C thermally aged material states of the studied TP316H material should exhibit the presence of minor secondary phases, namely, the Cr_23_C_6_-based carbides and Fe_2_Mo-based Laves phase. In the first estimate, the qualitative phase identification and differentiation were carried out by means of the back-scattered electron (BSE) contrast visualization of scanning electron microscopy images, which are highly sensitive regarding the average atomic number of a phase. Thus, the phases with great differences in their average atomic numbers, such as Cr_23_C_6_-based carbides and the Fe_2_Mo-based Laves phase can be reliably distinguished. Specifically, the Fe_2_Mo-based Laves phase particles with a much higher molecular mass (thanks to the presence of heavy Mo atoms) than Cr_23_C_6_-based carbides were identified on the BSE scanning electron microscopy images through their sharpness and bright contrast (Figure 6b). Conversely, the Cr_23_C_6_-based carbides, with a much lower average atomic number than the Fe_2_Mo-based Laves phase, show only weak grayish contrast (Figure 6a,b). Compared with the thermodynamic predictions, both Cr_23_C_6_-based carbides and Fe_2_Mo-based Laves phase particles were identified in only the 700 °C aged specimen (Figure 6b), whereas only the Cr_23_C_6_-based carbides were identified within the 450 °C aged specimen (Figure 6a). This result again indicates that the 450 °C aged specimen did not reach a material state corresponding to its thermodynamic equilibrium. This is due to the fact that the formation of Fe_2_Mo-based Laves-phase particles requires the diffusion of heavy Mo atoms at lower temperatures and proceeds with much slower kinetics compared to the diffusion rates at higher temperatures. Although the point EDX micro-analyses were carried out on central parts of the selected particles on the surfaces of prepared metallographic cross-sections, the obtained EDX spectra (Figure 6c,d) of individual minor phases can only be regarded in terms of qualitative phase differentiation, due to the fact of a higher electron beam interaction volume than that of the intended focused points of the EDX analyses. Thus, the Cr-rich particles and Mo-rich particles have been preliminarily indicated to be Cr_23_C_6_-based carbides and Fe_2_Mo-based Laves phase, respectively. 

**Figure 7:**
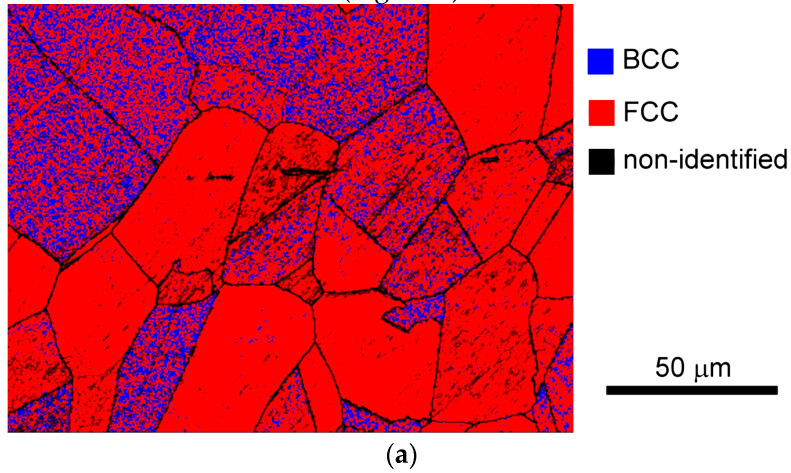

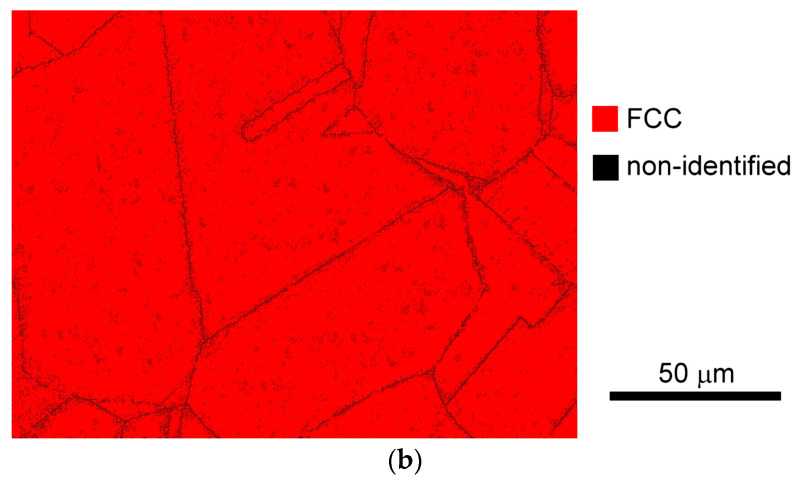
EBSD phase map for TP316H material aged at: (**a**) 450 °C for 5000 h and (**b**) 700 °C for 2500 h.

**Figure 8:**
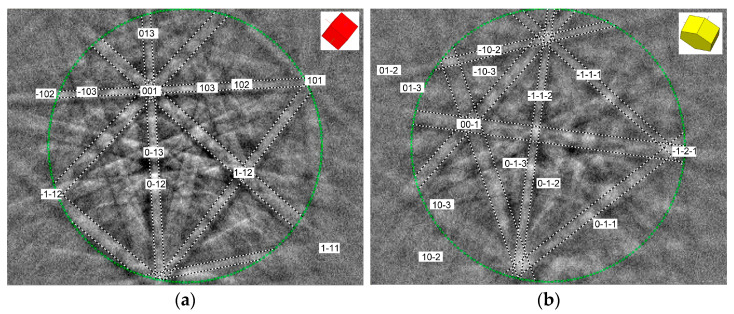
Indexed Kikuchi diffraction patterns of the minor phases in TP316H steel aged at 700 °C: (**a**) Cr_23_C_6_-based carbide with an FCC crystal structure of the NaCl type, (**b**) Fe_2_Mo-based Laves phase with HCP crystal structure of the MgZn_2_ type.

**Figure 9:**
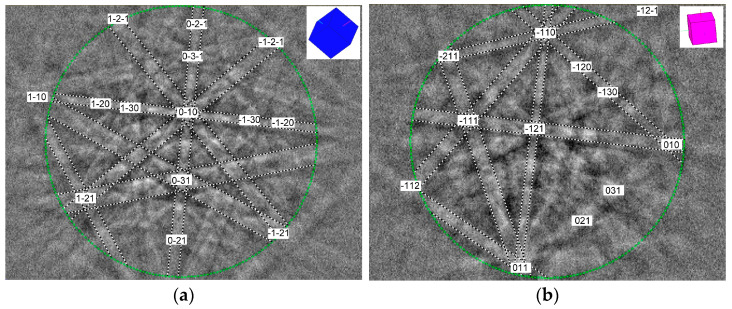
Indexed Kikuchi diffraction patterns of the major phases in TP316H steel aged at 450 °C: (**a**) austenitic Fe solid solution with an FCC crystal structure, (**b**) ferritic Fe solid solution with a BCC crystal structure.

For an unambiguous determination of individual phases in the studied heat-treated materials, EBSD crystallographic phase analyses were carried out by means of EBSD phase mapping and point Kikuchi diffraction patterns analyses. The overall EBSD phase map in Figure 7 shows that the matrix of the 450 °C thermally aged TP316H specimen consists of both austenite and ferrite (Figure 7a), whereas the matrix of the 700 °C thermally aged TP316H specimen consists of pure austenite (Figure 7b). These results agree fairly well with the performed thermodynamic predictions (Figure 3a and Figure 4) and XRD analyses (Figure 5). The non-identified regions in Figure 7 presumably represent the areas showing the occurrence of minor phases with other crystallographic characteristics. Due to the higher minor phase amounts and higher particle sizes of individual minor phases in the 700 °C thermally aged material (Figure 6), only the 700 °C thermally aged specimen was subjected to point EBSD crystallographic analyses that were focused on the obtaining and evaluation of characteristic Kikuchi diffraction patterns for individual minor phases (Figure 8). However, for the sake of completeness, the major phases in the specimen aged at 450 °C were also characterized by means of Kikuchi diffraction patterns from the selected microstructural areas (Figure 9).

### 3.2. Aging and Hydrogenation Effects on Impact Toughness and Fracture Behavior

**Figure 10:**
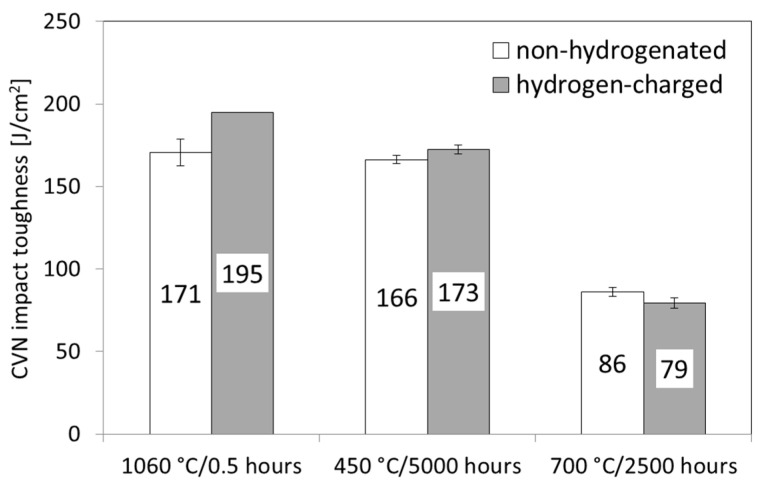
Dependence of the CVN impact toughness of TP316H steel on various material conditions with respect to the effect of high-temperature aging without or with subsequent room-temperature electrolytic hydrogen charging.

Figure 10 shows the dependence of room-temperature CVN impact toughness on previous annealing/aging material conditions and the subsequent application of room-temperature electrolytic hydrogen charging.

The results show that the samples’ initial soft-annealed material state exhibits the highest CVN impact toughness compared to the CVN impact toughness values for TP316H steel exposed to long-term isothermal aging expositions. Moreover, the initial soft-annealed material state shows, after subsequent electrochemical hydrogen charging, an even higher CVN impact toughness than that in non-hydrogenated material conditions. This hydrogen-toughening effect was also observed in our former study [36] and it has been ascribed to the hydrogen-enhanced twinning-induced plasticity (TWIP) effect. The TWIP ductilization behavior of hydrogen-charged TP316H steel has been supported by microstructural observations indicating a higher proportion of the deformation twinning mechanism in hydrogen-charged material compared to the non-hydrogenated material [36]. This conclusion agrees with numerous findings published in other works dealing with high-alloyed steels and high-entropy alloys with an FCC crystal structure [38,39,40,41,42,43,44,45,46,47,48]. Thus, from the viewpoint of the acting toughening mechanism, it has been generally accepted that the hydrogen-enhanced TWIP effect, including hydrogen-facilitated deformation nano-twinning, seemed to be the most likely hydrogen-induced ductilization mechanism [38,39,40,41,42,43,44,45,46,47,48]. Murakami et al. [47] reported the highly improved fatigue resistance of hydrogen-charged 304 and 316L stainless steels and concluded that the observed behavior was as a result of the interplay between two competitive roles of hydrogen, namely, the dislocation pinning and enhancement of dislocation mobility. Moreover, it should be noted that there are crucial differences between the characteristics of hydrogen behavior in FCC- and BCC-structured metals. The BCC-based metallic systems possess much lower hydrogen solubility at room temperature (e.g., about 1 wppm H for X70 ferritic-pearlitic pipeline steel [49]) compared to the FCC-structured metallic systems (e.g., around 100 wppm for 304 austenitic stainless steel [50]). As reported by Hickel et al. [51], the hydrogen solute occupies tetrahedral interstitial sites (T-sites) in the BCC crystal lattice, whereas in FCC-structured metals, the hydrogen solute prefers octahedral interstitial sites (O-sites) [52,53,54]. The overall number of T-sites in BCC crystal structures is lower than that of the O-sites in FCC crystal structures, resulting in lower hydrogen solubility in BCC iron than that in FCC iron [55]. The described differences between the BCC and FCC crystal structures in terms of hydrogen solute represent the preferential interstitial sites and also represent the origin of the higher hydrogen diffusivity in BCC metals compared to that in FCC metals, which is specifically due to the shorter diffusion distances between the nearest BCC T-sites and those of the FCC O-sites [54,56]. At room temperature, the hydrogen diffusivity in pure BCC iron is approximately 3.2 × 10^-6^ cm^2^/s, whereas the hydrogen diffusivity in pure FCC iron is only about 1.7 × 10^-16^ cm^2^/s [57]. In particular, the lower hydrogen diffusivity in FCC-structured materials is considered the crucial reason for their higher hydrogen embrittlement resistance compared to BCC-structured metallic materials [58].

**Figure 11:**
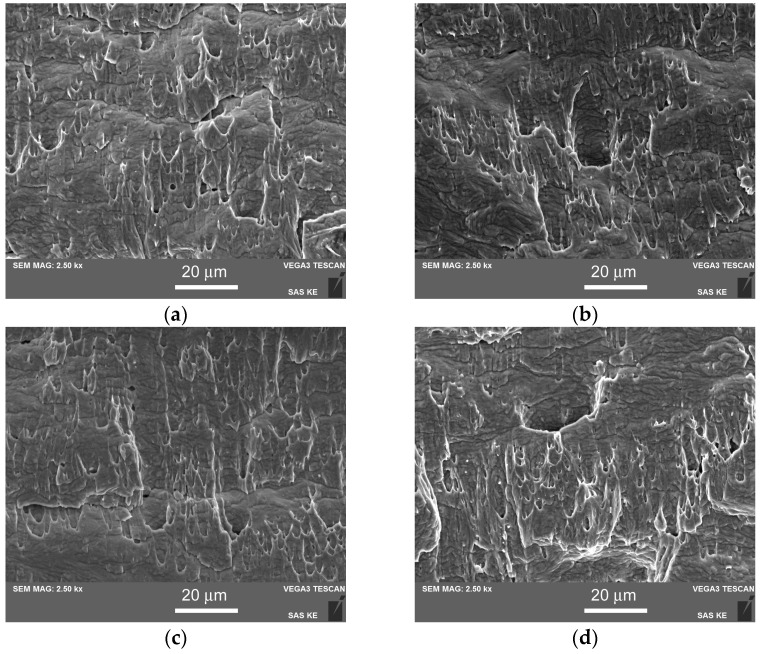

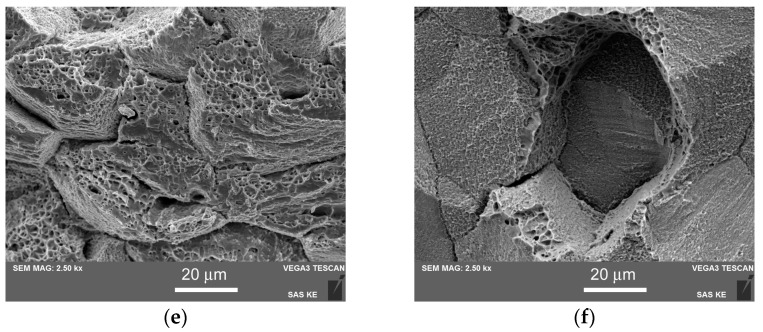
SEM fractographs corresponding to the individual material conditions of the studied TP316H stainless steel: (**a**) soft-annealed and non-hydrogenated, (**b**) soft-annealed and hydrogen-charged, (**c**) 450 °C/5000 h, non-hydrogenated, (**d**) 450 °C/5000 h, hydrogen-charged, (**e**) 700 °C/2500 h, non-hydrogenated, (**f**) 700 °C/2500 h, hydrogen-charged.

After performing an evaluation of the results of the Charpy impact bending tests, the subsequent fractographic characterization of broken test specimens was carried out in order to correlate the obtained properties with acting fracture micro-mechanisms. Figure 11 shows the recorded SEM fractographs corresponding to the individual material conditions of the studied TP316H stainless steel. In the initial soft-annealed material conditions, both the non-hydrogenated (Figure 11a) and hydrogen-charged (Figure 11b) TP316H materials exhibit ductile dimple tearing fracture micro-mechanisms, which correlate well with the correspondingly high values of impact toughness. The shallow elongated dimples are aligned in the direction of the tensile component of complex bending loading. Similar ductile dimple fractures are also observed for both the non-hydrogenated and hydrogen-charged TP316H materials aged at 450 °C (Figure 11c,d). This fracture behavior of the material aged at 450 °C is in accordance with its having similar microstructural characteristics to those of the initial material in the solution-annealed material condition. In contrast, the materials aged at 700 °C exhibit, under both the non-hydrogenated and hydrogen-charged material conditions, brittle fracture behavior that is governed by intergranular decohesion (Figure 11e,f). However, the intercrystalline areas are clearly decorated with ductile dimple features, indicating the localized micro-plasticity behavior that partially mitigates the material’s thermal embrittlement.

To make some relative quantitative comparisons of the effects of thermal aging and electrolytic hydrogen charging on the brittle-fracture resistance of the studied material under various material conditions, the embrittlement index (EI) can be calculated according to the following equation:

(1) $${\mathrm{EI}}_{\mathrm{CVN}}=\frac{{CVN}_{0}-{CVN}_{x}}{{CVN}_{0}}\;\times 100\%$$

where CVN_0_ and CVN_x_ are the values of the Charpy V-notch impact toughness of two individual material states [36]. The subscripts “0” and “x” refer to the states that are selected as initial and final, respectively. The values of the embrittlement index calculated according to Equation (1), using mean CVN values that are related to individual material conditions, are listed in Table 2.

From Table 2, it follows that after hydrogen charging and the subsequent Charpy impact bending test, the initial solution-annealed material (Row 1) exhibits the lowest EI value, which indicates that it has the highest hydrogen embrittlement resistance. Moreover, the negative EI value indicates an even more considerable hydrogen toughening effect. This result has been attributed to the hydrogen-enhanced twinning-induced plasticity (TWIP) deformation mechanism of the austenitic solid solution. In contrast, both thermally aged material states (Rows 2 and 3) exhibit more positive EI values compared to the initial solution-annealed state (Row 1), which indicates their lower hydrogen embrittlement resistance. The slightly negative EI value of the hydrogenated material aged at 450 °C (Row 2) still indicates some slight hydrogen-toughening effect, whereas the material aged at 700 °C (Row 3) shows a positive EI value, indicating a certain small hydrogen embrittlement of the aged material. The observed suppression of the hydrogen-toughening effect in thermally aged materials (Rows 2 and 3) compared to the initial solution-annealed (precipitation-free) material state (Row 1) is likely caused by the thermally induced precipitation of the secondary phase precipitates, which act like irreversible hydrogen traps, further resulting in decreasing the amount of diffusible hydrogen within the austenitic matrix. Thus, the hydrogen facilitation of the toughening TWIP effect in the austenitic matrix of thermally aged materials is also suppressed. Nevertheless, the studied material shows very good hydrogen embrittlement resistance in both aging conditions. The pure effect of thermal aging at 450 °C and 700 °C (i.e., without subsequent hydrogenation) of the TP316H material (Rows 4 and 5) characterizes its resistance against thermal embrittlement. It can be seen that the material aged at 450 °C does not exhibit any significant thermal embrittlement, whereas the material aged at 700 °C shows a significant degree of thermal embrittlement. Superposition of the effects of thermal aging and hydrogen charging (Rows 6 and 7) demonstrates the overall brittle-fracture resistance of the aged material states when subjected to subsequent electrolytic hydrogenation. It can be concluded that in the currently applied aging and hydrogenation conditions, the material aged at 450 °C shows very good brittle-fracture resistance (Row 6), whereas the material aged at 700 °C exhibits a notable deterioration of its resistance against brittle fracture. However, it should be noted that the major reason for the overall brittle-fracture resistance degradation of the 700 °C aged material is its thermally induced embrittlement due to the precipitation of the brittle Fe_2_Mo-based Laves phase. The hydrogen embrittlement resistance of both thermally aged materials is satisfactory. The obtained results clearly demonstrate that by an evaluation of the hydrogen embrittlement resistance of structural materials exposed to long-term high-temperature aging expositions, the separate evaluation and interpretation of individual contributing effects (i.e., thermal aging and hydrogen charging) provide a better understanding of overall material behavior. From a practical point of view, the obtained results indicate that the studied material is prone to thermal embrittlement but without any significant changes to its hydrogen embrittlement resistance. Our future work will, therefore, be focused on the possible rejuvenation heat treatment of the thermally aged TP316H material in correlation with its hydrogen embrittlement resistance.

## 4. Summary and Conclusions

In this work, the individual and superposition effects of thermal aging and electrolytic hydrogenation were studied on TP316H stainless steel. The obtained results are summarized in the following main conclusions:

- In the material aged at 700 °C for 2500 h, the precipitation behavior included the formation of densely distributed intergranular and intragranular secondary phase particles, specifically Cr_23_C_6_-based carbides and the Fe_2_Mo-based Laves phase. However, aging at 450 °C for 5000 h resulted in a much less pronounced precipitation of fine, mostly intergranular Cr_23_C_6_-based carbides.
- The matrix of the 700 °C aged material was formed of austenitic solid solution with an FCC crystal structure. Conversely, in the material aged at 450 °C, the additional formation of BCC-structured ferritic phase was found.
- The initial solution-annealed material exhibited high impact toughness under both the non-hydrogenated and hydrogen-charged conditions. The hydrogen-enhanced TWIP effect resulted in even higher CVN impact toughness, compared with the initial non-hydrogenated material. In contrast, both the thermally aged materials exhibited lower hydrogen embrittlement resistance, which was likely attributable to hydrogen trapping effects at the precipitate/matrix interfaces, leading to a reduced TWIP effect in the austenitic phase.
- The results of the impact toughness tests correlated well with the microstructural observations. The impact toughness deterioration of the “700 °C/2500 h” material state was predominantly caused by thermal embrittlement due to the precipitation of the intermetallic Fe_2_Mo-based Laves phase, occurring mainly on the grain boundaries. Conversely, the “450 °C/5000 h” material state did not show the precipitation of the brittle particles of the Laves phase within the timescale of the present investigation. Thus, thermal aging at 450 °C for 5000 h did not significantly affect impact toughness, whereas thermal aging at 700 °C for 2500 h resulted in significant thermal embrittlement.
- Regardless of the hydrogen charging application, fractographic observations after the Charpy impact bending tests revealed ductile dimple tearing fracture micro-mechanisms in both the solution-annealed and “450 °C/5000 h” thermally aged test specimens. In contrast, the fracture surfaces of the “700 °C/2500 h” thermally aged test specimens exhibited intergranular decohesion under both non-hydrogenated and hydrogen-charged conditions. The observed dimples on the surfaces of the intercrystalline fracture areas indicate the occurrence of micro-plastic behavior.

## Figures and Tables

**Table 1:** Elemental chemical composition in wt.% of the investigated TP316H stainless steel.

Material	C	Si	Mn	Cr	Mo	Ni	Fe
TP316H	0.052	0.51	1.77	16.76	2.05	11.13	rest

**Table 2:** Embrittlement index for the individual material states of TP316H steel.

Row	0	x	EI (0, x) [%]
1	1060 °C/0.5 h	1060 °C/0.5 h +H	−14.0
2	1060 °C/0.5 h + 450 °C/5000 h	1060°C/0.5 h + 450 °C/5000 h +H	−4.2
3	1060 °C/0.5 h + 700 °C/2500 h	1060°C/0.5 h + 700 °C/2500 h +H	8.1
4	1060 °C/0.5 h	1060°C/0.5 h + 450 °C/5000 h	2.9
5	1060 °C/0.5 h	1060 °C/0.5 h + 700 °C/2500 h	49.7
6	1060 °C/0.5 h	1060 °C/0.5 h + 450 °C/5000 h +H	−1.2
7	1060 °C/0.5 h	1060 °C/0.5 h + 700 °C/2500 h +H	53.8

Rows 1, 2, and 3—hydrogen embrittlement, Rows 4 and 5—thermal embrittlement, and Rows 6 and 7—superposition of thermal and hydrogen embrittlement.

## Data Availability

Data is contained within the article.
